# Chocolate, Air Pollution and Children's Neuroprotection: What Cognition Tools should be at Hand to Evaluate Interventions?

**DOI:** 10.3389/fphar.2016.00232

**Published:** 2016-08-11

**Authors:** Lilian Calderón-Garcidueñas, Vanessa San Juan Chávez, Nora B. Vacaseydel-Aceves, Raymundo Calderón-Sánchez, Edgar Macías-Escobedo, Carmen Frías, Marcela Giacometto, Luis Velasquez, Renata Félix-Villarreal, Jessie D. Martin, Christopher Draheim, Randall W. Engle

**Affiliations:** ^1^Biomedical Sciences, University of MontanaMissoula, MT, USA; ^2^Universidad del Valle de MéxicoCiudad de México, Mexico; ^3^Psychology, Universidad Autónoma de CoahuilaSaltillo, Mexico; ^4^Universidad AnáhuacMérida, Mexico; ^5^Facultad de Medicina, Universidad Andrés BelloSantiago de Chile, Chile; ^6^Universidad PanamericanaCiudad de México, Mexico; ^7^School of Psychology, Georgia Institute of TechnologyAtlanta, GA, USA

**Keywords:** air pollution, Alzheimer, children, chocolate, Mexico City, neuroinflammation, neuroprotection, working memory

## Abstract

Millions of children across the world are exposed to multiple sources of indoor and outdoor air pollutants, including high concentrations of fine particulate matter (PM_2.5_) and ozone (O_3_). The established link between exposure to PM_2.5_, brain structural, volumetric and metabolic changes, severe cognitive deficits (1.5-2 SD from average IQ) in APOE 4 heterozygous females with >75 − < 94% BMI percentiles, and the presence of Alzheimer's disease (AD) hallmarks in urban children and young adults necessitates exploration of ways to protect these individuals from the deleterious neural effects of pollution exposure. Emerging research suggests that cocoa interventions may be a viable option for neuroprotection, with evidence suggesting that early cocoa interventions could limit the risk of cognitive and developmental concerns including: endothelial dysfunction, cerebral hypoperfusion, neuroinflammation, and metabolic detrimental brain effects. Currently, however, it is not clear how early we should implement consumption of cocoa to optimize its neuroprotective effects. Moreover, we have yet to identify suitable instruments for evaluating cognitive responses to these interventions in clinically healthy children, teens, and young adults. An approach to guide the selection of cognitive tools should take into account neuropsychological markers of cognitive declines in patients with Alzheimer's neuropathology, the distinct patterns of memory impairment between early and late onset AD, and the key literature associating white matter integrity and poor memory binding performance in cases of asymptomatic familial AD. We highlight potential systemic and neural benefits of cocoa consumption. We also highlight Working Memory Capacity (WMC) and attention control tasks as opened avenues for exploration in the air pollution scenario. Exposures to air pollutants during brain development have serious brain consequences in the short and long term and reliable cognition tools should be at hand to evaluate interventions.

## Background

Increasing evidence links two key effects of air pollution (oxidative stress and neuroinflammation), to developmental neurotoxicity and neurodegenerative disease, particularly Alzheimer's disease (AD) (Blass and Gibson, 1991; Calderón-Garcidueñas et al., 2002, 2008a,b,c, 2012a,b, 2015a,b,c,d,e,f,g; Block and Calderón-Garcidueñas, 2009; Smith et al., 2010; Levesque et al., 2011a,b, 2013; Bolton et al., 2012; Oppenheim et al., 2013; Costa et al., 2014; Jung et al., 2015; Naini and Soussi-Yanicostas, 2015; Yao et al., 2015).

Children are exposed to significant amounts of complex mixtures of air pollutants, and their developing brains are at high risk for deleterious effects (Annavarapu and Kathi, 2016; Forns et al., 2015; Miller et al., 2016; Yorifuji et al., 2016). Sources of air pollutants include common environmental pollutants, molds, and fine particulate matter (PM_2.5_) (Querol et al., 2008; Molina et al., 2010; Múgica et al., 2010; Vega et al., 2010; Lippmann et al., 2013; Amato et al., 2014; Miller et al., 2016). PM_2.5_ is particularly dangerous for children's health (Bilenko et al., 2015), and factors such as particle size range, geographic location, source category, residency within a city, season, and socioeconomic status, all influence the impact on adverse health effects (Calderón-Garcidueñas and Torres-Jardón, 2012; Hajat et al., 2015). Systemic inflammation and increased concentrations of potent vasoconstrictors (i.e., endothelin-1, ET- 1) are critical features of children's exposure to the pollution (Calderón-Garcidueñas et al., 2007). Further, this inflammatory response correlates with cumulative exposures to PM_2.5_ (as well as total outdoor exposure hours), and occurs in conjunction with sustained inflammation of the upper and lower respiratory tracts and endothelial dysfunction (Calderón-Garcidueñas et al., 2003, 2007, 2008b).

Our work has centered specifically on Mexico City children, as they are exposed to a significant amount of pollution daily, including concentrations above the current US standards for ozone, and fine particulate matter < 2.5 μm in diameter (Molina et al., 2010). These life-long exposures are very significant in terms of oxidative stress, neurotoxicity, and neurodegeneration (Calderón-Garcidueñas et al., 2008a, 2009, 2012a, 2013a).

A crucial paper focused on the interaction between gender, BMI and APOE 4 makes a key observation: Gender, BMI and APOE influence children's cognitive responses to air pollution and glucose is likely a key player. We have described APOE 4 heterozygous females with >75 − < 94% BMI percentiles have the highest risk of severe cognitive deficits (1.5-2 SD from average IQ). These young females need gender-targeted health programmes to improve their cognitive responses. These are the females at the highest risk for the developing of Alzheimer and thus the need for early multidisciplinary intervention strategies (Calderón-Garcidueñas et al., 2016).

A recent paper by Jung et al. (2015), is highly relevant to the massive exposure of millions of people to high concentrations of **air pollutants** in megacities: an estimated 211% higher risk for AD per increase of 10.91 ppb in O_3_, and a 138% risk of increase of AD per increase of 4.34 μg/m^3^ in PM_2.5._ The study was done in a cohort of 95,690 individuals' age ≥ 65, over a 9 year follow-up period. Jung et al.'s findings strongly suggest long-term exposure to O_3_ and PM _2.5_ above the current US EPA standards are associated with increasing the risk of AD.

Key concept 1 **Air pollutants and alzheimer's risk**

Long-term exposure to ozone (O_3_) and fine particulate matter (PM _2.5_) above the current US EPA standards is associated with increased risk of AD (Jung et al., 2015).

A key consequence of air pollution exposure is neuroinflammation (Campbell et al., 2009; Levesque et al., 2011a,b, 2013). In urban children, our work has shown a significant frontal lobe imbalance in key genes for inflammation, oxidative stress, innate and adaptive immune responses, cell proliferation, and apoptosis (Calderón-Garcidueñas et al., 2012a). Moreover, otherwise clinically healthy Mexico City children exhibit cognition deficits, brain metabolic, structural and volumetric changes, and the neuropathological and cerebrospinal fluid (CSF) hallmarks of AD and Parkinson's diseases i.e., tau hyperphosphorylation with pre-tangles, amyloid beta42 (Aβ42) plaques, low CSF Aβ42, and misfolded α-synuclein accumulation (Calderón-Garcidueñas et al., 2008a, 2011, 2012a, 2013a, 2015a).

Extensive literature supports human and animal, breakdown of the nasal/olfactory blood-brain-barrier, alveolar-capillary, and intestinal barriers, as well as the brain expression of detrimental genes associated to urban air pollution (Thomson et al., 2007; Gerlofs-Nijland et al., 2010; Villarreal-Calderon et al., 2010; Bolton et al., 2012; Calderón-Garcidueñas et al., 2012a, 2013c, 2015g; Bergin and Witzmann, 2013; Carson et al., 2013; Kish et al., 2013; Ljubimova et al., 2013; Win-Shwe et al., 2014; Rossner et al., 2015; Tsamou et al., 2016). Specifically, inductively coupled plasma mass spectrometry for metal analysis and real time PCR in frontal samples of Mexico City children and young adults showed higher concentrations of metals associated with PM: manganese (*p* = 0.003), nickel and chromium (*p* = 0.02) along with higher frontal COX2 mRNA (*p* = 0.008) and IL1β (*p* = 0.0002) and COX2 (*p* = 0.005) olfactory bulb indicating neuroinflammation. Olfactory bulb DNA repair genes changes correlated with frontal combustion-related metals, suggesting that PM-metal neurotoxicity plays a key role in brain damage in young urbanites (Calderón-Garcidueñas et al., 2013c). Upregulated gene network clusters involved in inflammation, immunity, differentiation, cell growth, tumorigenesis, and apoptosis, including IL1, NFκB, TNF, IFN, and TLRs are described in frontal samples of MC young urbanites v clean air controls (Calderón-Garcidueñas et al., 2012a). While a 15-fold frontal down-regulation of the prion-related protein (PrP(C)) with important roles for neuroprotection, neurodegeneration, and mood disorder states is also seen in highly exposed subjects (Calderón-Garcidueñas et al., 2012a). Upregulation of inflammatory genes is not restricted to supratentorial structures, the dorsal vagal complex (DVC) is also a target (Villarreal-Calderon et al., 2010). Cyclooxygenase-2 (COX-2), interleukin 1 beta (IL-1β), and CD14 messenger RNA (mRNA) were quantified after 4, 8, and 16 months of exposure in mice target brain regions. After 16 months of exposure to the MC atmosphere v controls, the DVC exhibited significant inflammation in MC mice (COX-2 and IL-1β *P* < 0.001) along with the olfactory bulb upregulation of CD14 (*P* = 0.002) and significant DVC imbalance in genes for antioxidant defenses, apoptosis, and neurodegeneration. Similar gene cluster changes are seen upon exposure to diesel PM or a combination of PM +ozone (Gerlofs-Nijland et al., 2010; Levesque et al., 2011a,b, 2013; Ljubimova et al., 2013).

Neuroinflammation, cognitive deficits, weight gain, memory function and maternal performance based on the impaired gene expressions in the hippocampus and hypothalamus and placental epigenetic changes with fetal impact are described for specific pollutants such as diesel, as well as a combination of complex mixtures of PM2.5 (Bolton et al., 2012; Win-Shwe et al., 2014; Tsamou et al., 2016).

Of particular concern in environments with high concentrations of ultrafine particulate matter (UFPM, nanosize particles < 100 nm), is that UFPM ends up in contact with the vascular endothelium where it can induce damage (Gehr et al., 2011; Sharma and Sharma, 2012; Sharma et al., 2013; Karmakar et al., 2014; Ucciferri et al., 2014). There is robust evidence that nanosize particles can increase endothelial paracellular permeability *in vitro*, and induce endothelial TJ opening (Sharma et al., 2013; Karmakar et al., 2014; Ucciferri et al., 2014). Exposure to different size particulate matter (including nano size particulate matter), is associated with production and deposit of misfolded protein aggregates (amyloid, alpha synuclein, hyperphosphorilated tau), oxidative stress, cell damage, and death in susceptible neuronal populations (Qin et al., 2007; Hartz et al., 2008; Levesque et al., 2011a,b, 2013; Mushtaq et al., 2015; Parveen et al., 2015; Tian et al., 2015). The frontal white matter is an early and key target of air pollution exposures in young Mexico City residents (Calderón-Garcidueñas et al., 2015f). Major light and electron microscopic findings include: leaking in capillaries and small arterioles, thickening of cerebrovascular basement membranes with small deposits of amyloid, patchy absence of the perivascular glial sheet, enlarged Virchow-Robin spaces and nanosize particles (20–48 nm) in endothelium, basement membranes, mitochondria, axons and dendrites (Calderón-Garcidueñas et al., 2015f). In canine studies, tight junctions, a key component of the neurovascular unit (NVU) are abnormal in Mexico City vs. rural control dogs (χ^2^ < 0.0001), and white matter perivascular damage is significantly worse in MC dogs (*p* = 0.002) (Calderón-Garcidueñas et al., 2015f). The integrity of the NVU, an interactive network of vascular, glial and neuronal cells is compromised in **MC young residents**.

Key concept 2 **Mexico city children's brains**

Extensive neuroinflammation, breakdown of the neurovascular unit, oxidative stress, and hallmarks of Alzheimer disease pathology are present in the brains of Mexico City children and young adults with chronic yearlong exposures to high levels of O_3_ and PM_2.5_.

The issue of early oxidative stress is particularly important in pediatric cohorts, given findings suggesting that “a vicious downward spiral involving the interactions between mitochondrial dysfunction and oxidative stress contributes to the initiation and/or amplification of reactive oxygen species that is critical to the pathogenesis of AD” (Wang X. et al., 2014).

Neuroprotection is critical for pediatric and young adult populations residing in highly polluted environments and given our previous experience using cocoa and dark chocolate to decrease neuroinflammation and improve cognition in young Mexico City residents (Villarreal-Calderon et al., 2010; Calderón-Garcidueñas et al., 2013b), we undertook a review of potential systemic and CNS benefits of cocoa consumption. We also explored the use of cognitive assessments, and their ability to identify early, subtle changes in cognitive performance.

## Neuroprotection: chocolate, an ancient friend and a new player

Cocoa health benefits have been appreciated for centuries (Latif, 2013; Sokolov et al., 2013; Grassi et al., 2015a,b). The health benefits of cocoa are numerous and wide ranging. Not the least of which include positive impacts on pathways associated with neurodegeneration (Schini-Kerth, 2014; De la Monte, 2014; Jumar and Schmieder, 2016). Because of the multifaceted nature of AD pathology, neuroprevention should include targeting multiple potential pathophysiological mechanisms (Dubner et al., 2015). A fundamental issue in using cocoa as a neuroprotector, is to take into account factors that determine absorption, metabolism, and excretion of cocoa flavonols, all of which are key aspects of evaluating potential benefits (Cifuentes-Gómez et al., 2015). Moreover, the presence of flavonols in complex food matrices, including their interactions with other nutrients/non-nutrients, significantly influence their absorption in the intestinal lumen, as well as their transfer across the GI barrier (Cifuentes-Gómez et al., 2015). For example, in pediatric and young adult populations with high exposure to air pollutants, the transfer of flavonols across the GI barrier could be compromised by the extensive tight junction (TJs) damage (Calderón-Garcidueñas et al., 2015g), and the production of TJs autoantibodies (Calderón-Garcidueñas et al., 2015c). Additionally, carbohydrate interactions are critical for the flavonol GI absorption; subsequently, in children, the absorption and metabolism results of the combination of cacao with sugar vs. sucrose, milk (of varying fat content), or lactose intolerance can't be dismissed (Dehkordi et al., 1995; Schramm et al., 2003; Schroeter et al., 2003; Roura et al., 2008; Rodriguez-Mateos et al., 2012; Cifuentes-Gómez et al., 2015).

The issue of brain bioavailability is also important. The passage of the active metabolites through the blood-brain-barrier (BBB), and the detection of certain metabolites in specific brain regions depend on multiple factors. These may include: the type of metabolite (i.e., flavonols, flavonones), lipophilicity of each compound, use of pure compounds, and the integrity of the BBB (Abd El Mohsen et al., 2002; Youdim et al., 2004; Milbury and Kalt, 2010; Faria et al., 2014).

Strategies designed to explain the mechanisms by which brain-bioavailable flavonols may beneficially influence cognitive deficits need to be made available (Wang et al., 2010; Wang J. et al., 2014; Dubner et al., 2015). Several recent papers review the direct, and indirect, mechanisms potentially associated with cocoa's beneficial neural effects (Desideri et al., 2012; Sokolov et al., 2013; Jumar and Schmieder, 2016; Grassi et al., 2015a,b). Overall, evidence suggests that cardiovascular effects are important in neuroprotection, specifically with regard to the impact on endothelial dysfunction (Jumar and Schmieder, 2016). To this end, cocoa flavonols improve vasodilatory capacity in high and low cardiovascular risk subjects (Heiss et al., 2003; Ferri et al., 2015; Jumar and Schmieder, 2016; Sansone et al., 2015). Given the obesity epidemic, and comorbid health problems in Mexico and the USA, cocoa and cocoa flavonoids may positively affect the pathophysiological mechanisms involved in insulin resistance and endothelial dysfunction, with potential benefits in the prevention of cardiometabolic diseases (Grassi et al., 2015a,b). The issue of insulin resistance and diabetes mellitus is important because epidemiological studies support that type 2 diabetes is a major contributor to AD risk (Cermakova et al., 2015; Ramos-Rodríguez et al., 2015; Sato and Morishita, 2015), and growing evidence supports the key concept that *Alzheimer's disease is a metabolic disease, mediated by impairments in brain insulin responsiveness, glucose utilization, and energy metabolism, which ultimately lead to increased oxidative stress, inflammation, and worsening of insulin resistance* (De la Monte, 2014).

Thus, lowering blood pressure, inhibiting platelet aggregation, increasing the bioavailability of nitric oxide and reducing inflammatory mediators are critical factors for optimizing cardiovascular health (Ostertag et al., 2010; Ferri et al., 2015; Jumar and Schmieder, 2016; Gormaz et al., 2016) as well as the central nervous system (Fisher et al., 2006; Sorond et al., 2010).

There is a long list of beneficial neural effects of cocoa including: epigenetic mechanisms targeting multiple classes of chromatin writer-reader-eraser proteins related to histone acetylation-methylation and DNA methylation, improvement of memory and learning through enhanced dentate gyrus function, and decreased anxiety with elevated hippocampal monoamine and Brain Derived Neurotrophic Factor (BDNF) levels (Van Praag et al., 2007; Brickman et al., 2014; Stringer et al., 2015; Declerck et al., 2016). However, researchers agree that knowledge of the ultimate action of cocoa flavonols on the human brain remains limited (Sokolov et al., 2013). Moreover, there is a paucity of cocoa flavonols studies on mood, cognitive and cardiovascular health research in young people (Francis et al., 2006; Field et al., 2011; Scholey and Owen, 2013; Massee et al., 2015), and very little research on the effects of cocoa in urban children, a population which could benefit immensely from cocoa interventions (Calderón-Garcidueñas et al., 2013b).

If we are going to use **cocoa as a neuroprotectant** in otherwise healthy urban children and young adults, more research is needed, including:

1. To define cocoa cognitive effects in the target urban highly exposed population and the clear air counterparts.
2. To define vascular effects, both systemic, and cerebral.
3. To define antidiabetic, antistress, antiobesity, and anti-inflammatory effects.
4. To define the health risks from sustained administration of cocoa, including weight gain, alteration of lipid profiles and allergies.

Key concept 3 **Cocoa and neuroprotection**

Cocoa's beneficial neural effects include the impact on endothelial function and their positive effects on insulin resistance. There is a knowledge gap in the potential beneficial and detrimental effects of long term administration of cocoa in clinically health young urbanites.

Equally important to the potential for cocoa to serve as a neuroprotector, is the access to a quantifiable measure of its cognitive impact. As such, repeated cognitive testing in teens and young adults is essential.

Of key importance to select the right cognitive tools is the documented evidence that clinically healthy Mexico City children and young adults exhibit the neuropathological and cerebrospinal fluid (CSF) features associated with AD, i.e., tau hyperphosphorylation with pre-tangles, amyloid beta42 (Aβ42) diffuse plaques, and low CSF Aβ42 (Calderón-Garcidueñas et al., 2008a, 2011, 2012a, 2013a, 2015a). Thus, an approach to guide the selection of cognitive tools should take into account neuropsychological markers of cognitive declines in patients with Alzheimer's neuropathology, the distinct patterns of memory impairment between early and late onset AD, and the key literature associating white matter integrity and poor memory binding performance in cases of asymptomatic familial AD.

## Neuropsychological markers of cognitive decline in asymptomatic familial AD, cognitively health older adults with AD neuropathology and patients with early AD

There is agreement that the pathological hallmarks of Alzheimer disease are present before the onset of symptoms significant to trigger a clinical diagnosis of mild cognitive impairment (MCI) or dementia (Driscoll et al., 2006; Twamley et al., 2006; Grober et al., 2008; Howieson et al., 2008; Price et al., 2009; Riley et al., 2011; Sperling et al., 2011; Beach et al., 2012; Monsell et al., 2014; Hassenstab et al., 2015; Pettigrew et al., 2015). A particularly interesting study from Hassenstab et al. (2015) examined 314 elderly individuals *cognitively healthy at study entry*. These individuals died within 2 years of their last minimum two visits for neuropsychological performance evaluation and received a diagnosis of low to high AD neuropathological change. *The main cognitive abnormal findings at baseline* involved nearly all domains of cognition, with pronounced effects on executive functioning, language and episodic memory. Monsell et al., reported subjects with AD neuropathological change but without MCI or dementia, having subtle decline in the attention/working memory (WM) domain. Hassenstab et al. (2015) suggested *attention/WM might be the earliest subtle neuropsychological domain to be affected in the preclinical phase of AD*.

In the work of Chapuis et al. (2016) 76 people with MRI were rated for microangiopathy, hippocampal and parietal atrophy and a gradient of fronto-parietal atrophy. A standardized neuropsychological battery for attention, language, praxis, parietal function, visuoconstructive function, verbal and visual memory, WM, and frontal executive function were used. Working memory (WM) deficits proved to be a reliable battery among the three groups characterized by microangiopathy, hippocampal atrophy and parietal atrophy. It is key to emphasize that microangiopathy indeed is associated with executive disorders and impaired WM (Au et al., 2006; Schmidt et al., 2007; Son et al., 2012), because indeed Mexico City youngsters have significant white matter alterations in the neurovascular unit, with clear evidence of microangiopathy (Calderón-Garcidueñas et al., 2015f).

The white matter integrity as measured by diffusion tensor magnetic resonance imaging is critical to explain poor memory binding performance in symptomatic carriers of the E280A mutation of the PSEN1 gene (Parra et al., 2015). Interestingly, asymptomatic young carriers of the mutation, ages 24–43 year differed from controls only in the short-term memory binding task. Parra's paper is critical for our review because frontal white matter alterations have an impact on WM (Owen, 2000; Prabhakaran et al., 2000; Sala and Courtney, 2007). Alterations in myelin, oligodendrocytes, axonal degeneration and vascular pathology are key factors to play a role in frontal white matter to account for WM deficits (Englund and Brun, 1990; Sjobeck et al., 2005; Bartzokis et al., 2007). Equally relevant to Mexico City young residents is the knowledge that white matter synaptic disruption precedes both white matter tract anomalies and neurodegeneration (Alix and Domingues, 2011). We fully agree with Parra et al., that “*different memory binding functions may be affected by different white matter events.”*

The issue of impairments of WM and inhibitory control are absolutely critical in the context of air pollution exposures and WM deficits. Eye-tracking tasks deficits are seen in people with early dementia (Crawford et al., 2005, 2013, 2015; Crawford and Highm, 2016). Saccadic eye movements (rapid gaze shifts) are under the control of a network of cortical and subcortical connections involved in WM and inhibitory control (IC) (Crawford and Highm, 2016). The anti-saccade task (AST) is the most common used IC paradigm that includes a central inhibitory component with a high proportion of corrective eye movements following the inhibition failures (Crawford et al., 2013). The corrective eye movements are less frequent in people with AD (Crawford et al., 2013), so it remains to be seen whether **highly exposed Mexico City youngsters** are impaired on the antisaccade task and if impaired WM can coexist with an intact IC, as suggested by Crawford and Highm (2016).

High levels of eye movement distractibility are seen in the early stages of AD (Crawford and Highm, 2016), such a deficiency in the ability to inhibit irrelevant information will significantly impact memory. Also relevant, prominent non-memory domains, including executive functions and visuoconstructional abilities are common in early onset AD (EOAD) (Joubert et al., 2016).

Mexico City children have significantly lower CSF Aβ_1−42_ concentrations vs. their matched clean air controls (Calderón-Garcidueñas et al., 2015a). This poses a key question in the open exposed urban population: are the CSF levels of Aβ_1−42_ relevant for cognition? The information so far is not clear when cognitively normal middle age and older adults are examined. Pettigrew et al. work suggests *CSF A*β_**1-42**_ *is not significantly associated with cognition*, while robust associations between biomarkers of amyloid pathology and episodic memory are found (Pike et al., 2007; Villemagne et al., 2011; Hedden et al., 2012; Kantarci et al., 2012; Pettigrew et al., 2015). Interestingly, Pettigrew et al. also reported that APOE4 status was not directly associated with cognitive performance, an association we have reported in our Mexico City highly exposed children' cohorts (Calderón-Garcidueñas et al., 2015b,e).

Key concept 4 **Detection of children at high risk**

Our efforts to design a neurocognitive battery for highly exposed Mexico City young residents capable to detecting subtle cognition changes in specific domains, is based on the literature reports focusing in the asymptomatic stages of familiar AD and in the profile of cognitive deficits reported for clinically cognitively intact middle and older adults with AD neuropathology and in patients with early AD.

## Working memory capacity and inhibitory control

It is important that serious thought be given to the measurement of cognition in studying the effects of air pollution. Considerations should include: (1) measures of cognition should be as common across studies as possible, (2) measures should be as domain-general as possible—meaning they should reflect processes and capabilities that are common to many different cognitive tasks, (3) the measures and the constructs they reflect should be moderately well understood in cognitive theory, and (4) they should be relatively easily measured across different environments and in different ages. Working memory and its related constructs meets all these criteria. Working memory capacity (WMC) can be defined as the ability to store or keep active information temporarily for use in ongoing tasks, to update, transform, and modify that information in ongoing processing, and, when necessary to disengage or to inhibit recently activated information (Engle and Kane, 2004). Working memory capacity is highly correlated with fluid intelligence (Gf), the biological and largely inherited aspect of intelligence. One important reason for this correlation seems to be that both types of measures rely on the ability to control ones attention, a capability often referred to as Executive Attention (EA). Extensive work under the rubric of cognitive control (e.g., WMC/Gf/EA) has shown that these capabilities are important to a huge range of educational and real-world cognitive tasks from reading and listening comprehension to complex learning and problem solving (Engle and Kane, 2004; Kane et al., 2004). Thus, the assessment of these constructs can be useful, and even necessary, to be able to make conclusions about the role of pollution on cognitive performance of individuals in their own environment.

We have selected key WMC/Gf/EA tasks for exploring highly exposed teens and young adults in Mexico City including.

### Complex spans

Complex span tasks, the most commonly used measures of WMC have been shown to reflect strong relationships with real-world cognition (Daneman and Carpenter, 1980). We will work with two variations: operation and symmetry span that require test-participants to remember serially-presented items (e.g., letters, words, spatial locations). Each to-be-remembered item is followed by a processing task that has to be completed before the next item is shown and which serves to force attention shifts back and forth between the items to be remembered and the processing task. For the operation span task, a mathematical equation must be solved. For the symmetry span task, a picture must be judged as either symmetrical or non-symmetrical. After several pairs of items and processing tasks have been presented (generally 2–7), test-takers are to reconstruct the list of items in the order in which they were originally presented. Performance on complex span tasks is strongly predictive of a person's attention control abilities (Engle and Kane, 2004; Unsworth et al., 2009; Unsworth and Spillers, 2010; Hutchison, 2011). Key to the quest for subtle cognitive alterations in air pollution, complex span tasks best predict performance on complex cognition tasks (e.g., reading, listening, complex learning, and problem solving) as well as for emotional and behavioral self-control associated with control of inappropriate social behavior (Broadway et al., 2010). Thus, reductions in measures of cognitive control (e.g., WMC/Gf/EA) are associated with a wide range of socially relevant behaviors including, alcohol abuse and violence. Addiction behaviors and violence deserve study because are relevant potential consequences of the effects of pollution on cognitive control.

### Attention control tasks

There are two aspects of attention. One way to think about attention is that various events capture our attention. Those events could be externally generated such as a loud sound or internally generated such as the thought of something that happened to you recently. However, the more important aspect of attention for present purposes is how we exert control over our attention and direct it toward a task we are attempting to perform. We can think of that as EA. A good example of this is our tendency to mind wander. It is now clear that people who are measured higher on tasks of cognitive control are much less likely to have their attention captured and to mind wander when performing a task (Kane and McVay, 2012).

There are several reliable and valid tasks of attention control.

#### One of them is the antisaccade task

In a common version of this task, subjects are faced with a computer screen with a fixation cross in the center of the screen and two boxes 11 degrees of visual angle to each side of the fixation. They are to stare at the fixation cross and at some point one of the boxes will flicker. When they happens they are to immediately shift their attention to the box on the opposite side of the screen where a letter (either O or Q) will be presented for a very short duration (150 ms) after which it is masked. The subject must identify which letter was presented. The problem is that evolution has prepared all organisms with a brain to have their attention captured by the flickering box because the flicker affords movement in the natural world and things that move can eat you or you can eat them. This tasks requires that individuals resist that natural temptation and force their attention to the opposite side of the screen (Kane et al., 2001). Antisaccade performance is highly associated with WMC and Gf and predicts many different real-world behaviors including self-control.

#### Flanker task

The arrow flanker task based on work by Eriksen and Eriksen (1974) in which subjects are to identify the middle character of a 5 character string. For example, in the string of character X X → XX, the subject would be required to press a key indicating a right facing as opposed to left facing arrow. In the following string of characters, ←← → ←←, the response is the same but the subject must resist the strong automatic activation of the response associated with the competing and more numerous arrows pointing left. As in the antisaccade task, performance on this task is strongly predictive of a wide range of cognitive and emotional behaviors (Kane et al., 2004).

### General fluid intelligence tasks

Modern theories of intelligence generally distinguish two types of intelligence: Crystallized intelligence is culturally-derived knowledge such as vocabulary and other things that we have learned from our environment and fluid intelligence, our ability to reason and solve problems which are novel to us. Fluid intelligence is thought to be the biological and genetically derived aspect of intelligence and is the most domain-general. Therefore, it is the aspect of intelligence that is of most interest to our studies of the effects of air pollution. Measures of fluid intelligence, abbreviated as Gf are:

1. Raven's advanced progressive matrices (Raven, 1990). Participants see a 3 − 3 matrix in which 8 abstract figures have been placed. Participants chose which of several options belonged in the ninth box. Ten minutes are given to complete 18 problems. The dependent variable is the number of correct responses.
2. Letter sets (Ekstrom et al., 1976). Participants see five sets of four-letter sequences. They need to discover the rule that is common to four of the sets and then indicate which set does not belong. Five minutes are given to complete 30 problems. The dependent variable is the number of correct responses.
3. Number series (Thurstone, 1938). Participants see a series of numbers and select which of several options complete the series. Five minutes are given to complete 15 problems. The dependent variable is the number of correct responses.

All WM tasks have strong, direct, relationships to attention control. It is difficult to maintain to-be-remembered items while alternately performing secondary processing tasks (Shipstead et al., 2014, 2015). Attention control requires engagement in the service of organizing the contents of primary memory (Engle, 2002; Kane et al., 2007).

The relationship between attention control, fluid intelligence, and WM capacity is of great relevance to people exposed to environmental air pollution. Any effect that attention control has on novel reasoning is realized through an effect on memory and also fluid intelligence. The issue is then to define in highly exposed individuals the capability for cognitive control as reflected by tasks of WM capacity, fluid intelligence, and EA.

Since the individual's ability to resist attention capture explains a large portion of both WM capacity and its relationship to higher cognitive abilities (Shipstead et al., 2014), the critical point in the results of highly exposed air pollution cohorts vs. clean matched low pollution controls will be to define the interaction of attention control and memory. This in turn, will give us a picture of the impact of environmental factors upon both WM capacity and fluid intelligence in a developing brain. Equally important in terms of the risk of developing Alzheimer, will be the identification of individuals at the highest risk, defined by the lower scores in the tasks measuring WMC and attention control. We already know of the strong relationship between chronic exposure above the standards of ozone and PM_2.5_ and risk of newly diagnosed Alzheimer disease as clearly shown by Jung et al. (2015).

Since the prevalent consensus is that AD starts decades before clinical diagnosis, and given that these at-risk children and young adults already exhibit WM capacity deficits reflective of well documented systemic and neural pathological changes, the delineation of the relative temporal trajectories of cognitive measures will be key in the evaluation of cocoa administration or any other intervention. The application of the selected tasks, would allow the creation of a framework for the early identification of neurodegeneration biomarkers related to air pollution exposures. Current knowledge should give us the opportunity to intervene in young urban populations, independent of what specific air pollutants are responsible within the complexity of the atmospheric chemistry and the microenvironments of each subject.

As such, an essential goal of future work will be to elucidate the extent of beneficial and potential detrimental effects of chronic cocoa administration to children and young adults and whether it provides real benefits with respect to reducing cognitive impairment.

Exposures to air pollutants during brain development have serious brain consequences in the short and long term and reliable cognition tools should be at hand to identify individuals at their highest risk and to evaluate neuroprotective interventions.

## Author contributions

LC planned and directed the study, wrote the manuscript, edited and formatted the manuscript. VS planned the study, contribute to writing the manuscript. NV contributed to the writing of the paper. EM contributed to the writing of the paper. RC contributed to the writing of the paper. CF contributed to the writing of the paper. MG contributed to the writing of the paper. LV contributed to the writing of the paper. RF contributed to the writing of the paper. JM contributed to the writing of the paper. CD contributed to the writing of the paper. RE contributed to the writing of the paper.

### Conflict of interest statement

The authors declare that the research was conducted in the absence of any commercial or financial relationships that could be construed as a potential conflict of interest.
